# Supplementary material for: Uncoupling Protein 3 Catalyzes the Exchange of C4 Metabolites Similar to UCP2
Source: Biomolecules. 2023 Dec 22;14(1):21. doi: 10.3390/biom14010021 (PMC10813146; doi:10.3390/biom14010021)
Supplement: Supplementary file 1 [file biomolecules-14-00021-s001.zip › biomolecules-2664646-supplementary.pdf]

SUPPORTING INFORMATION

Uncoupling protein 3 catalyzes the exchange of C4 metabolites similar to UCP2.

Jürgen Kreiter[1,2,§], Tatiana Tyshchuk[1,§], Elena E. Pohl[1,*]

[1] Institute of Physiology, Pathophysiology and Biophysics, University of Veterinary Medicine, A-1210 Vienna, Austria

[2] Present address: Institute of Molecular and Cellular Physiology, Stanford Medical School, Stanford, CA, USA

[3] Current address: Ludwig Boltzmann Institute for Experimental and Clinical Traumatology in the AUVA Trauma Research Centre, A-1200 Vienna, Austria

§ Equally contributed.

*Corresponding author. Email: elena.pohl@vetmeduni.ac.at

```
mUCP3   MVGLQPSEVPPTTVVKFLGAGTAACFADLLTFPLDTAKVRLQIQGENPG----AQSVQYR   56
mUCP2   MVGFKATDVPPTATVKFLGAGTAACIADLITFPLDTAKVRLQIQGESQGLVRTAASAQYR   60
mANT1   -----MGDQALSFLKDFLAGGIAAAVSKTAVAPIERVKLLLQVQHASKQ---ISAEKQYK   52

mUCP3   GVLGTILTMVRTEGPRSPYSGLVAGLHRQMSFASIRIGLYDSVKQFYTPKGADHSSV---   113
mUCP2   GVLGTILTMVRTEGPRSLYNGLVAGLQRQMSFASVRIGLYDSVKQFYT-KGSEHAGI---   116
mANT1   GIIDCVVRIPKEQGFLSFWRGNLANVIRYFPTQALNFAFKDKYKQIFLGGVDRHKQFWRY   112

mUCP3   -AIRILAGCTTGAMAVTCAQPTDVVKVRFQAMIRLGTGGERKYRGTMDAYRTIAREEGVR   172
mUCP2   -GSRLLAGSTTGALAVAVAQPTDVVKVRFQAQARA--GGGRRYQSTVEAYKTIAREEGIR   173
mANT1   FAGNLASGGAAGATSLCFVYPLDFARTRLAADVGKG-SSQREFNGLGDCLTKIFKSDGLK   171

mUCP3   GLWKGTWPNITRNAIVNCAEMVTYDIIKEKLLESHLFTDNFPCHFVSAFGAGFCATVVAS   232
mUCP2   GLWKGTSPNVARNAIVNCAELVTYDLIKDTLLKANLMTDDLPCHFTSAFGAGFCTTVIAS   233
mANT1   GLYQGFSVSVQGIIIYRAAYFGVYDTAKGMLPDPKNVHIIV—SWMIAQSVTAVAGLVSY-   229

mUCP3   PVDVVKTRYMNAP-----LGRYRSPLHCMLKMVAQEGPTAFYKGFVPSFLRLGAWNVMMF   287
mUCP2   PVDVVKTRYMNSA-----LGQYHSAGHCALTMLRKEGPRAFYKGFMPSFLRLGSWNVVMF   288
mANT1   PFDTVRRRMMMQSGRKGADIMYTGTLDCWRKIAKDEGANAFFKGAWSNVLRGM-GGAFVL   288

mUCP3   VTYEQLKRALMKVQVLRESPF   308
mUCP2   VTYEQLKRALMAAYQSREAPF   309
mANT1   VLYDEIKKYV-----------   298
```

Figure S1. Alignment of the amino acid sequences of murine UCP3, UCP2 and ANT1.
The highly conserved PX[DE]XX[KR] signature motif of mitochondrial carriers is marked in blue. The three amino acids involved in the putative substrate binding site in the central cavity of mUCP3, mUCP2 and mANT1 are colored in red.

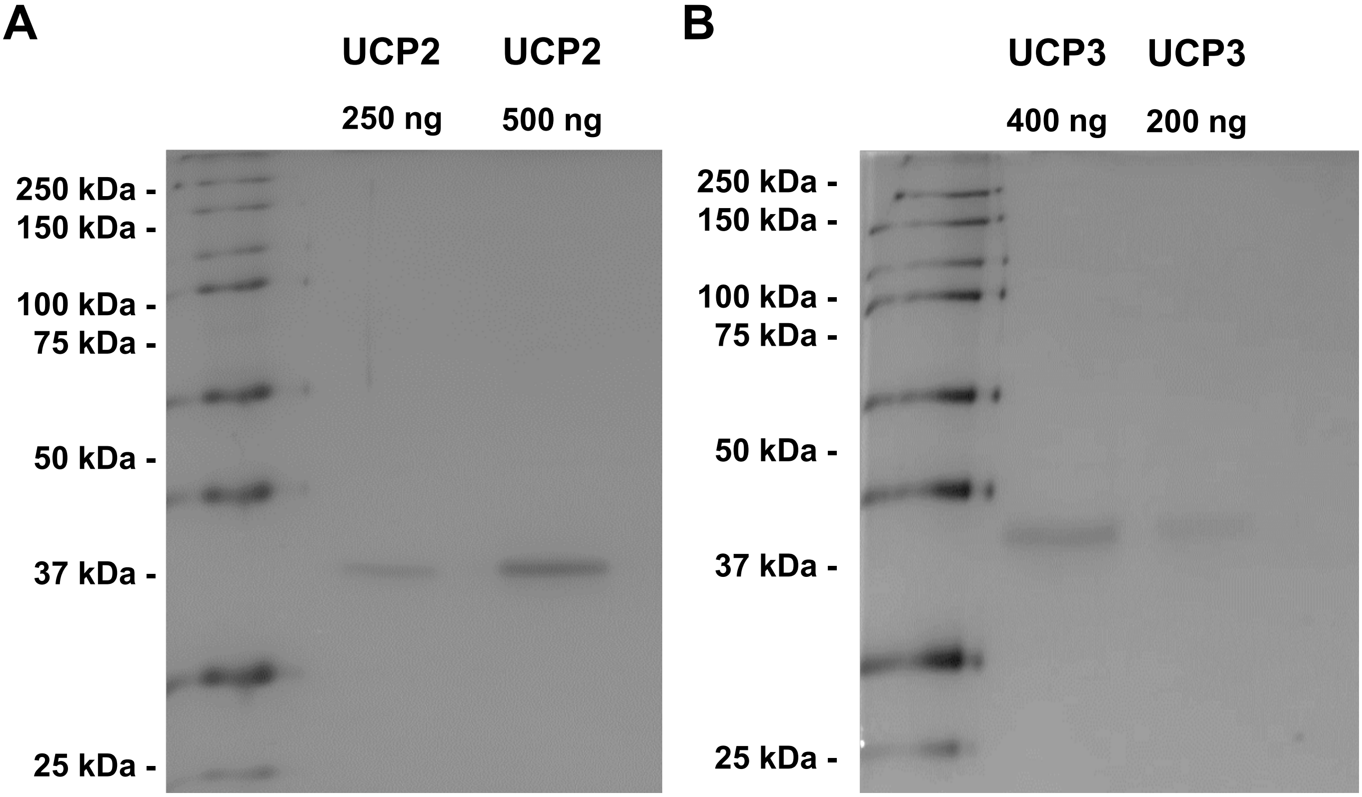

Figure S2. Representative silver staining of reconstituted UCP2 and UCP3.

For quality control, 250 ng or 500 ng of UCP2 (A) and 400 ng and 200 ng of UCP3 (B) contaioning proteoliposomes were loaded onto a 15% acrylamide gel and SDS-PAGE was conducted. Subsequently, proteins were visualized by silver staining.

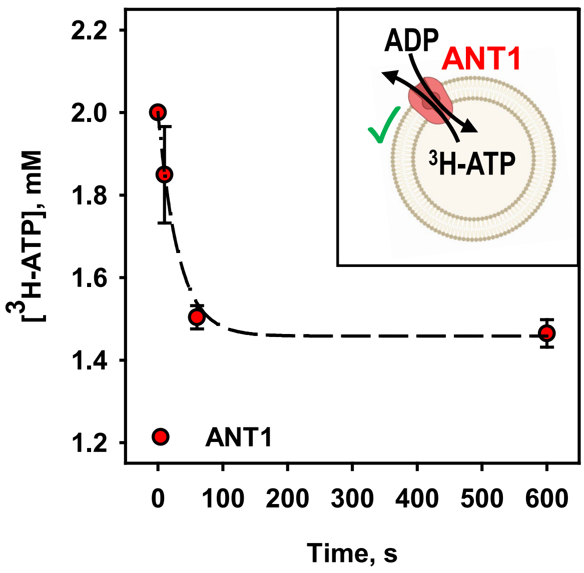

Figure S3. ANT1-mediated ADP/ATP exchange as a control for ANT1 activity.

Efflux of ATP from liposomes over time measured by remaining intraliposomal ^{3}H-ATP radioactivity of ANT1-containing liposomes. Concentration of intraliposomal ^{3}H-ATP and extraliposomal ADP was 2 mM. **Inset:** Experimental setup to test ADP/ATP exchange of ANT1.

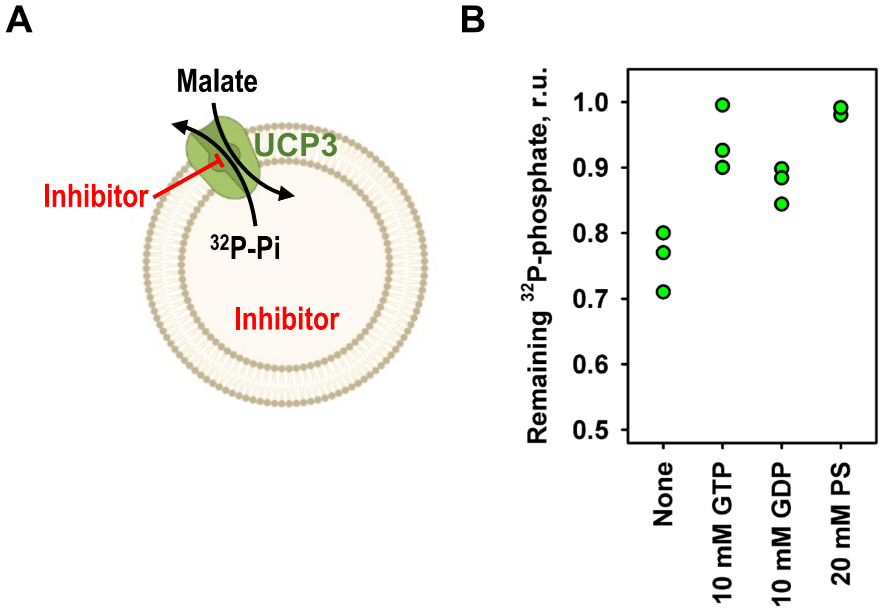

Figure S4. Inhibition of UCP3-mediated phosphate/malate exchange.

(A) Experimental setup to measure the exchange of ^{32}P-phosphate against malate in the presence of various inhibitors. **(B)** Remaining ^{32}P-phosphate in liposomes containing UCP3 after T = 600 s as the ratio to the initial ^{32}P-phosphate amount at T = 0 s. Concentration of ^{32}P-phosphate and malate was 2 mM, respectively. In the experiments, the lipid concentration was 4 mg/ml and the protein concentration was 4 µg/mg of lipid. Membranes were made of 45:45:10 mol% PC:PE:CL. Buffer solution contained 50 mM Na_2SO_4, 10 mM Tris, 10 mM MES and 0.6 mM EGTA at pH = 7.34 and T = 296 K.

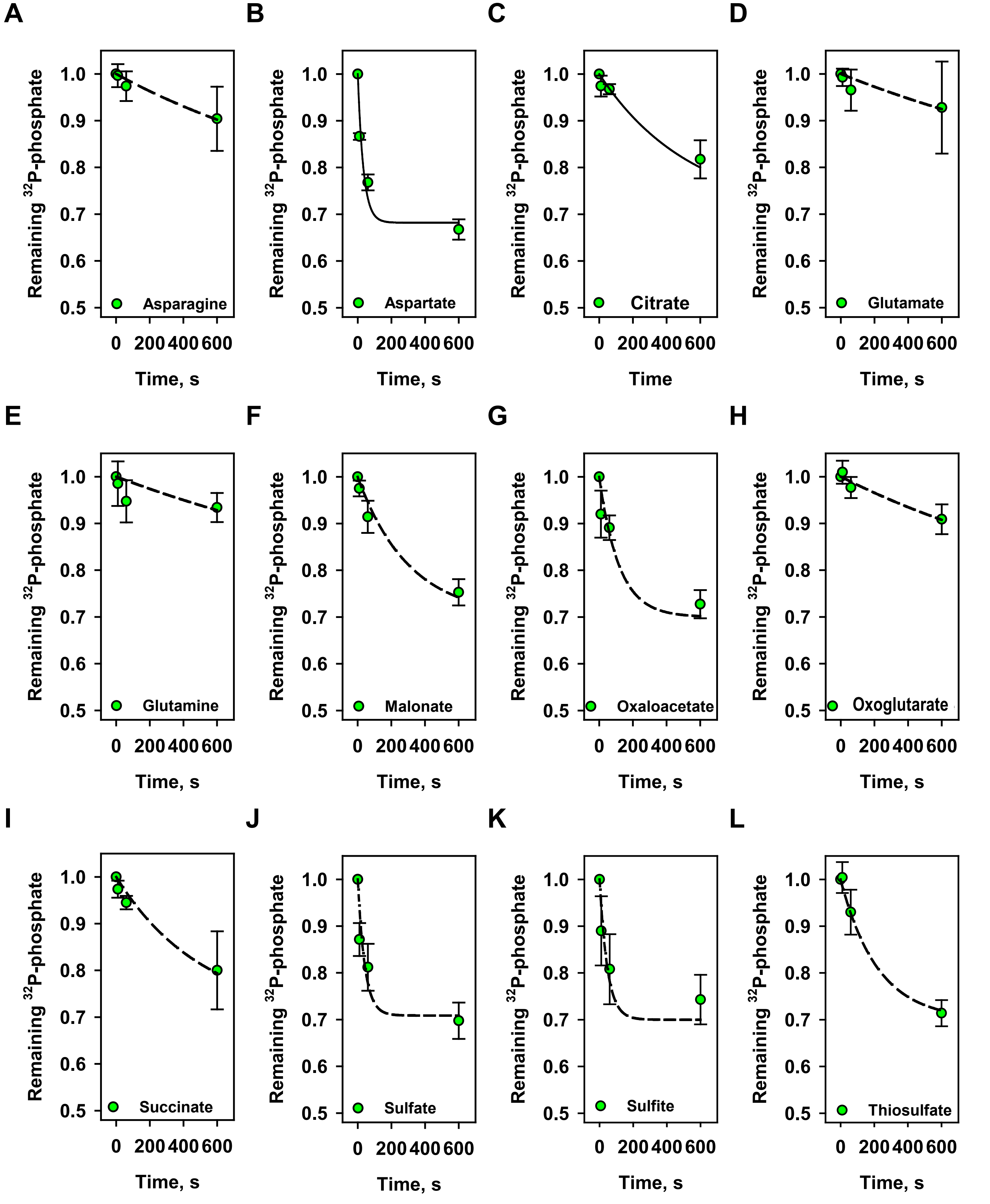

Figure S5. Evaluation of different substrates as possible metabolites transported by UCP3

Time course of intraliposomal phosphate concentration in the presence of UCP3 and extraliposomal asparagine (A), aspartate (B), citrate (C), glutamate (D), glutamine (E), malonate (F), oxaloacetate (G), oxoglutarate (H), succinate (I), sulfate (J), sulfite (K) or thiosulfate (L). In the experiments, the lipid concentration was 4 mg/ml and the protein concentration was 4 µg/mg of lipid. Membranes were made of 45:45:10 mol% PC:PE:CL. Buffer solution contained 50 mM Na_2SO_4, 10 mM Tris, 10 mM MES and 0.6 mM EGTA at pH = 7.34 and T = 296 K. Data are the mean ± SD of at least three independent experiments.

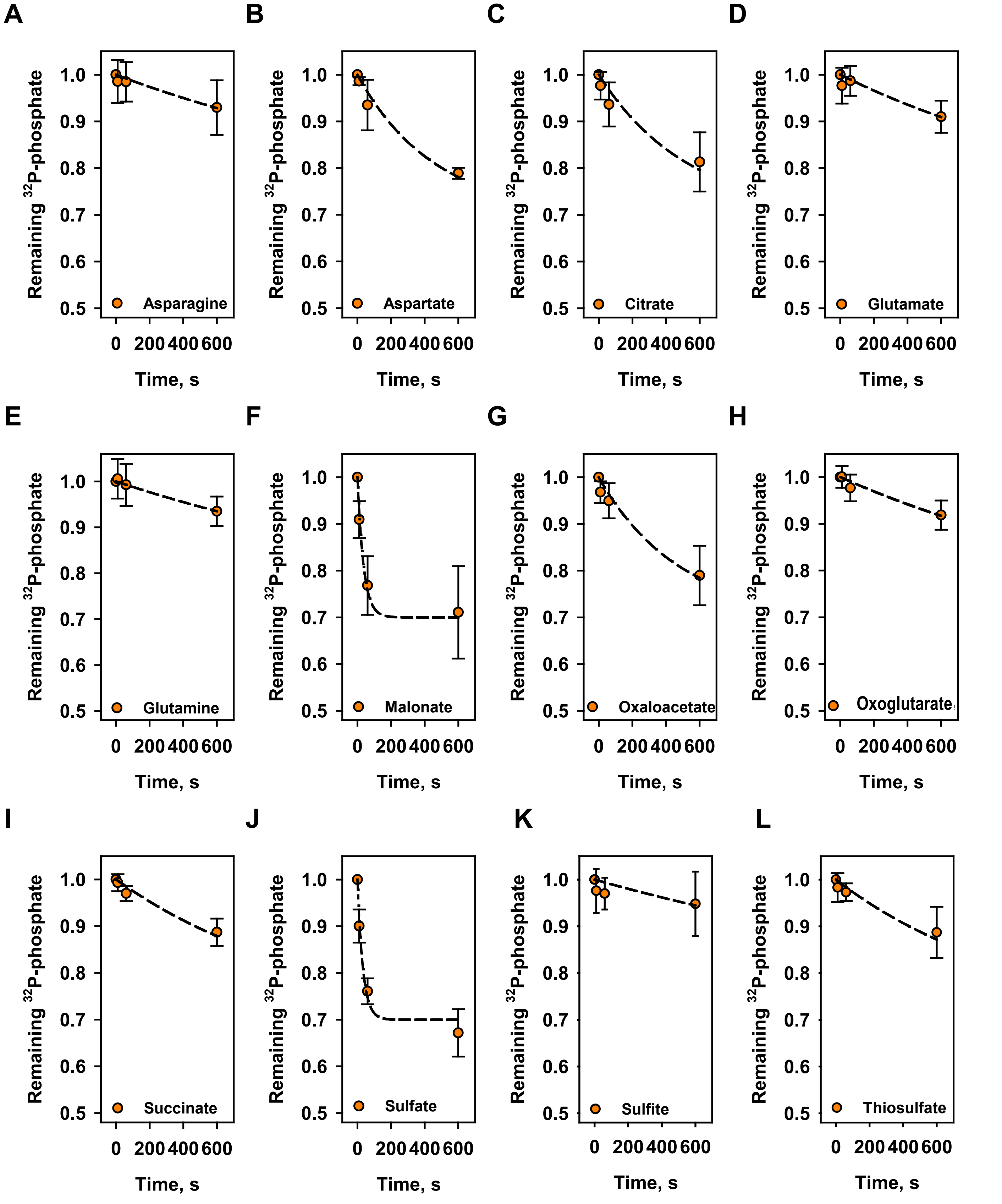

Figure S6. Evaluation of different substrates as possible metabolites transported by UCP2

Time course of intraliposomal phosphate concentration in the presence of UCP2 and extraliposomal asparagine (A), aspartate (B), citrate (C), glutamate (D), glutamine (E), malonate (F), oxaloacetate (G), oxoglutarate (H), succinate (I), sulfate (J), sulfite (K) or thiosulfate (L). In the experiments, the lipid concentration was 4 mg/ml and the protein concentration was 4 μg/mg of lipid. Membranes were made of 45:45:10 mol% PC:PE:CL. Buffer solution contained 50 mM Na_2SO_4, 10 mM Tris, 10 mM MES and 0.6 mM EGTA at pH = 7.34 and T = 296 K. Data are the mean ± SD of at least three independent experiments.

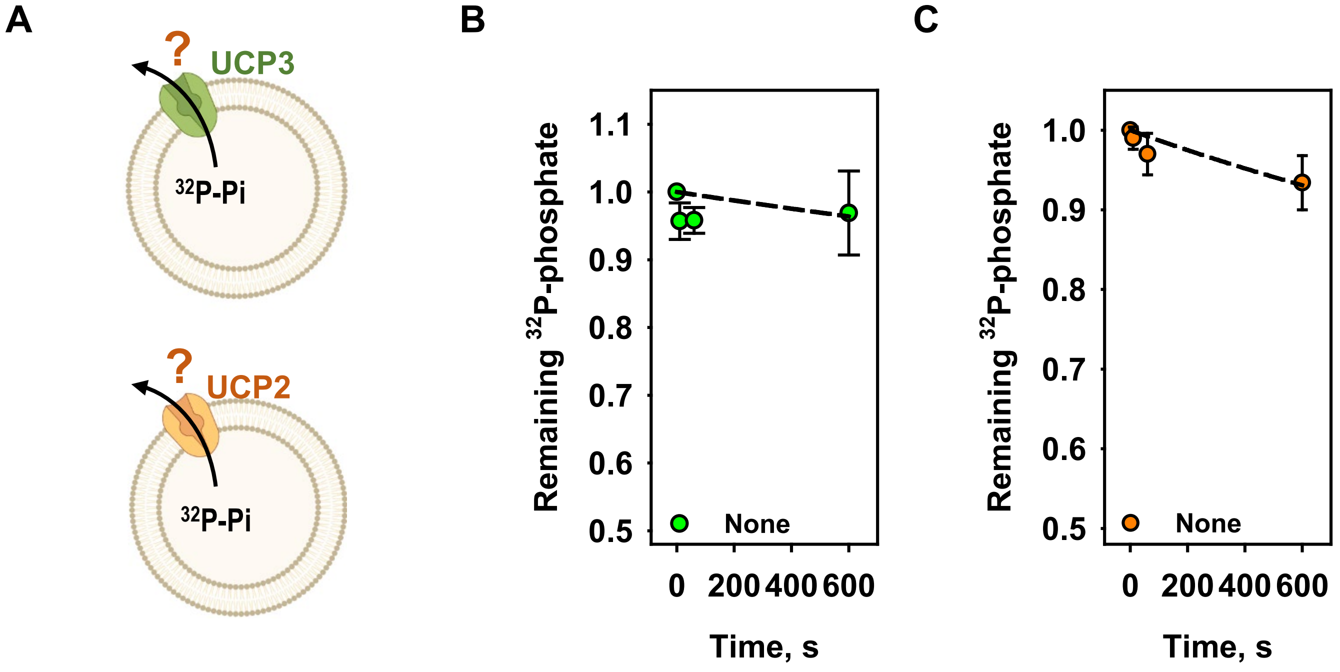

Figure S7. Uniport transport of phosphate of UCP3 and UCP2

(A) Release of intraliposomal ^{32}P-phosphate in the absence of an external substrate. **(B, C)** Time course of intraliposomal ^{32}P-phosphate efflux concentration in the presence of liposomes containing UCP3 **(B)** or UCP2 **(C)**. Concentration of ^{32}P-phosphate was 2 mM. In the experiments, the lipid concentration was 4 mg/ml and the protein concentration was 4 µg/mg of lipid. Membranes were made of 45:45:10 mol% PC:PE:CL. Buffer solution contained 50 mM Na_2SO_4, 10 mM Tris, 10 mM MES and 0.6 mM EGTA at pH = 7.34 and T = 296 K.

Table S1: Overview of transport rates of various mitochondrial carriers reconstituted into liposomes. Abbreviations are: cit – citrate, mal – malate, 2-oxog – oxoglutarate, Pi – phosphate, asp – aspartate, glu – glutamate and cys - cysteinsulfinate

Protein	Species	Transport rate, mmol/min/g protein	Substrates	Ref.
**Citrate carrier (CiC – SLC25A1)**	Rat	2.05 ± 0.09	cit/cit	[1]
		1.87 ± 0.11<br>2.06 ± 0.13	mal/cit	
		1.98 ± 0.16	mal/mal	
**Phosphate carrier (PiC – SLC25A3)**	Bovine	70 - 90	Pi/Pi	[2]
		30	Pi/OH^-	
**Dicarboxylate carrier (DiC – SLC25A10)**	Rat	6.4 – 6.7	mal/Pi	[3]
		7.3	mal/mal	
**Oxoglutarate carrier (OGC – SLC25A11)**	Bovine	2.96 - 22	oxog/oxog	[4] – [6]
		10.34 ± 0.37		[7]
**Aspartate/glutamate carrier 1 (AGC1 – SLC25A12)**	Human	25.5 – 40.5	asp/asp	[8]
		16.1 – 23.7	asp/glu	
		21.8	glu/glu	
		17.7 – 27.6	asp/cys	
		14.2	glu/cys	
**Aspartate/glutamate carrier 2 (AGC2 – SLC25A13)**		92.8 – 178.4	asp/asp	
		55.3 – 118	asp/glu	
		90.3	glu/glu	
		81.2 – 133.8	asp/cys	
		65.9	glu/cys	

[1] Bisaccia, F., De Palma, A., Prezioso, G., and Palmieri, F. (1990) Kinetic characterization of the reconstituted tricarboxylate carrier from rat liver mitochondria. Biochim. Biophys. Acta. 1019, 250–256.

[2] Stappen, R., & Krämer, R. (1993). Functional properties of the reconstituted phosphate carrier from bovine heart mitochondria: evidence for asymmetric orientation and characterization of three different transport modes. Biochimica et Biophysica Acta (BBA)-Biomembranes, 1149(1), 40-48.

[3] Indiveri, C., Prezioso, G., Dierks, T., Krämer, R., & Palmieri, F. (1993). Kinetic characterization of the reconstituted dicarboxylate carrier from mitochondria: a four-binding-site sequential transport system. Biochimica et Biophysica Acta (BBA)-Bioenergetics, 1143(3), 310-318.

[4] Indiveri C, Palmieri F, Bisaccia F, Krämer R (1987b) Kinetics of the reconstituted 2-oxoglutarate carrier from bovine heart mitochondria. Biochim Biophys Acta 890:310–318

[5] Indiveri C, Dierks T, Krämer R, Palmieri F (1991a) Reaction mechanism of the reconstituted oxoglutarate carrier from bovine heart mitochondria. Eur J Biochem 198:339–347

[6] Fiermonte G, Walker JE, Palmieri F (1993) Abundant bacterial expression and reconstitution of an intrinsic membrane-transport protein from bovine mitochondria. Biochem J 294:293–299

[7] Smith, V. R., & Walker, J. E. (2003). Purification and folding of recombinant bovine oxoglutarate/malate carrier by immobilized metal-ion affinity chromatography. Protein expression and purification, 29(2), 209-216.

[8] Palmieri, L., Pardo, B., Lasorsa, F. M., Del Arco, A., Kobayashi, K., Iijima, M., ... & Palmieri, F. (2001). Citrin and aralar1 are Ca2+-stimulated aspartate/glutamate transporters in mitochondria. The EMBO journal, 20(18), 5060-5069.
